# Disruption of PD-1 Enhanced the Anti-tumor Activity of Chimeric Antigen Receptor T Cells Against Hepatocellular Carcinoma

**DOI:** 10.3389/fphar.2018.01118

**Published:** 2018-10-01

**Authors:** Xingliang Guo, Hua Jiang, Bizhi Shi, Min Zhou, Honghong Zhang, Zhimin Shi, Guoxiu Du, Hong Luo, Xiuqi Wu, Yi Wang, Ruixin Sun, Zonghai Li

**Affiliations:** ^1^State Key Laboratory of Oncogenes and Related Genes, Shanghai Cancer Institute, Renji Hospital, Shanghai Jiao Tong University School of Medicine, Shanghai, China; ^2^CARsgen Therapeutics, Shanghai, China

**Keywords:** hepatocellular carcinoma, immunotherapy, chimeric antigen receptor, PD-1, gene editing, CRISPR-Cas9

## Abstract

Cancer immunotherapy has made unprecedented breakthrough in the fields of chimeric antigen receptor-redirected T (CAR T) cell therapy and immune modulation. Combination of CAR modification and the disruption of endogenous inhibitory immune checkpoints on T cells represent a promising immunotherapeutic modality for cancer treatment. However, the potential for the treatment of hepatocellular carcinoma (HCC) has not been explored. In this study, the gene expressing the programmed death 1 receptor (PD-1) on the Glypican-3 (GPC3)-targeted second-generation CAR T cells employing CD28 as the co-stimulatory domain was disrupted using the CRISPR/Cas9 gene-editing system. It was found that, *in vitro*, the CAR T cells with the deficient PD-1 showed the stronger CAR-dependent anti-tumor activity against native programmed death 1 ligand 1-expressing HCC cell PLC/PRF/5 compared with the wild-type CAR T cells, and meanwhile, the CD4 and CD8 subsets, and activation status of CAR T cells were stable with the disruption of endogenous PD-1. Additionally, the disruption of PD-1 could protect the GPC3-CAR T cells from exhaustion when combating with native PD-L1-expressing HCC, as the levels of Akt phosphorylation and anti-apoptotic protein Bcl-xL expression in PD-1 deficient GPC3-CAR T cells were significantly higher than those in wild-type GPC3-CAR T cells after coculturing with PLC/PRF/5. Furthermore, the *in vivo* anti-tumor activity of the CAR T cells with the deficient PD-1 was investigated using the subcutaneous xenograft tumor model established by the injection of PLC/PRF/5 into NOD-scid-IL-2Rγ-/- (NSG) mice. The results indicated that the disruption of PD-1 enhanced the *in vivo* anti-tumor activity of CAR T cells against HCC, improved the persistence and infiltration of CAR T cells in the NSG mice bearing the tumor, and strengthened the inhibition of tumor-related genes expression in the xenograft tumors caused by the GPC3-CAR T cells. This study indicates the enhanced anti-tumor efficacy of PD-1-deficient CAR T cells against HCC and suggests the potential of precision gene editing on the immune checkpoints to enhance the CAR T cell therapies against HCC.

## Introduction

Hepatocellular carcinoma, the most predominant type of primary liver cancer, is one of the leading causes of cancer-related death and arouses global concern in recent years (Abdalla et al., 2018; Hu et al., 2018; Jin et al., 2018). Because most (more than 80%) of patients with HCC are diagnosed at a late stage of the disease, potentially curative therapies (including ablation, chemotherapy, proton beam therapy, chemoembolization, and targeted drug therapy) are often less effective and only extend the overall survival by a short time (Llovet et al., 2008; Gao et al., 2014; Xiang et al., 2015).

CAR T cells are genetically engineered T cells expressing an artificial recombinant receptor molecule (CAR) on the cell surface. The CAR combines antigen-binding domain [most commonly, a single-chain variable fragment (scFv) derived from the variable domains of antibodies] with the signaling domain of the TCRζ chain and additional costimulatory domain(s) from receptors such as CD28, OX40, and 4-1BB that promote the proliferation and survival of T cell, endowing T cells with MHC-independent target recognition and a fundamental antitumor advantage (Kuwana et al., 1987; Gross et al., 1989; Di and Li, 2016; June et al., 2018). With the unprecedented success of the CAR T cells in leukemia and lymphoma, a growing number of studies have focused on the treatment of solid tumors using the CAR-T technology (Bagley et al., 2018). Excitingly, it was found that T cells expressing GPC3-targeted CAR can efficiently kill GPC3-positive HCC cells (Gao et al., 2014). Furthermore, the relevant phase 1 clinical trial study (ClinicalTrials.gov identifier: NCT02395250) showed that autologous T cells bearing CAR that can specifically recognize GPC3 was safe and effective for patients with relapsed or refractory HCC (Zhai et al., 2017). Meanwhile, the phase 1 clinical trial (ClinicalTrials.gov identifier: NCT02541370) of CD133-directed CAR T cells for advanced HCC showed that the feasibility, controllable toxicities, and effective activities of the CAR T cells for treating the patients with CD133-positive HCC (Wang et al., 2018). Thus, adoptive cell therapy based on CAR-redirected T (CAR T) cells has been identified as an effective and promising strategy for the treatment of patients with HCC. However, the efficacy of CAR T cells in the solid tumor is prone to be affected due to the immunosuppressive tumor microenvironment [e.g., expression of inhibitory ligands programmed death 1 ligand (PD-L) 1/ligand 2 on tumor cells and surrounding tissues for the PD-1 of T cells], which impairs the function and persistence of adoptively transferred T cells (Leen et al., 2007; Rabinovich et al., 2007; Joyce and Fearon, 2015; Bagley et al., 2018). PD-1 is a prominent checkpoint receptor expressed on T cells following activation (Harvey, 2014). PD-1:PD-L1/L2 pathway plays an important role in dampening T cell response and increasing T cell susceptibility to apoptosis (Bardhan et al., 2016; Papaioannou et al., 2016). Fortunately, tumor-induced downregulation of T cell function can be reversed using immune checkpoint inhibitors that block PD-1-mediated signaling cascades and maintain T cell activation within the tumor microenvironment (Pardoll, 2012; Papaioannou et al., 2016), suggesting that the disruption of endogenous PD-1-mediated inhibitory signaling could be beneficial to the antitumor activity of CAR T cells.

The CRISPR/Cas system is an adaptive immune system in prokaryotes, and the CRISPR/Cas9 system has recently been exploited for genome engineering (Cong et al., 2013). Su et al. (2016) found CRISPR-edited T cells with deficient PD-1 showed the enhanced cytotoxicity on the PD-L1 expressing melanoma and gastric cells *in vitro*. Rupp et al. (2017) showed that CRISPR/Cas9-mediated PD-1 disruption enhances anti-tumor efficacy of human CAR T cells against myelogenous leukemia, but the target tumor cell expressing PD-L1 was artificially constructed by lentiviral transduction, and the efficacy on the native PD-L1 expressing tumor cells remains unclear. Ren et al. (2017) demonstrated that the disruption of PD-1 led to enhanced *in vivo* antitumor activity of CAR T cells against pancreatic cancer cell and B-cell precursor leukemia cells, while only the cells with high stable expression of PD-L1 artificially constructed by lentiviral transduction was used in leukemia model. Additionally, these studies employed the 4-1BBζ CARs rather 28ζ CAR. The CAR T cells employing different costimulatory domains shows differential antitumor activity and PD-1 expression (Carpenito et al., 2009; Guedan et al., 2014, 2018; Zhao et al., 2015). 28ζ CAR T cells usually showed stronger anti-tumor activities relative to BBζ CAR T cells, and BBζ CAR T cells often exhibited greater *in vivo persistence* compared with 28ζ CAR T, although the characteristics of *in vivo* expansion and persistence between 28ζ CAR T and BBζ CAR T cells were variant in different tumor models. Zhong et al. (2010) showed that 28ζ CAR T cells displayed stronger *in vitro* and *in vivo* anti-tumor activities, and superior *in vivo* expansion compared with BBζ CAR T cells in the prostate cancer model. Zhao et al. (2015) found, in acute lymphoblastic leukemia model, 28ζ CAR T cells showed similar *in vitro* cytotoxicity and stronger *in vivo* anti-tumor activity compared with BBζ CAR T cells, but BBζ CAR T cells showed greater persistence than 28ζ CAR T cells. Li et al. (2017) found 28ζ CAR T cells showed stronger *in vitro* cytotoxicities and similar *in vivo* anti-tumor activities against HCC compared with BBζ CAR T cells, although BBζ CAR T cells showed superior *in vivo* expansion, and preferentially produced Th1 cytokines (interferon γ/granulocyte macrophage colony-stimulating factor) in contrast to 28ζ CAR T cells to preferentially produce Th2 cytokines (interleukin-4/interleukin-10). Moreover, each different cancer has a different microenvironment associated with that malignancy (Hou et al., 2016; Ruvolo, 2016). Liver is characterized by the inherent immunosuppressive environment, and the PD-L1 expression was found on HCC and the majority of the liver myeloid-derived suppressor cells (Chen et al., 2016; Thorn et al., 2016). So far, it remains unclear for the effect of disruption of endogenous PD-1 on the antitumor activity of CAR T cells employing CD28 as the co-stimulatory domain against HCC.

In the present study, the endogenous PD-1 in the second-generation GPC3-targeted CAR T cells employing CD28 as the co-stimulatory domain was disrupted using the CRISPR-Cas9 gene-editing system. The *in vitro* and *in vivo* antitumor efficacy of PD-1-deficient CAR T cells against native PD-L1-expressing HCC and the effects of the CRISPR-mediated disruption of endogenous PD-1 on CD4 and CD8 subsets, and activation status of CAR T cells were studied.

## Materials and Methods

### Safety

Over the course of this study, the standard biosecurity and institutional safety procedures were followed for handling biohazards, biological select agents, toxins, and restricted materials or reagents.

### Cell Culture

Human HCC cell lines (GPC3-positive PLC/PRF/5 and GPC3-negative SK-HEP-1) (Gao et al., 2014) and human embryonic kidney (HEK) 293T cell line were obtained from the American Type Culture Collection. The GPC3-positive SK-HEP-1/GPC3 cell line was constructed by lentiviral transduction of SK-HEP-1 with Pwpt-GPC3 virus encoding human GPC3 in the previous study of our research group (Yu et al., 2018). All the cell lines were maintained in Dulbecco’s modified eagle medium (DMEM) (Gibco, United States) supplemented with 10% FBS (Gibco, United States). Peripheral blood mononuclear cells (PBMC) were obtained from Shanghai Blood Center. PBMC and the activated T cells were maintained in AIM-V medium (Gibco, United States) supplemented with 2% human AB serum (ABS, Gemini Bioproducts, United States) and 500 U/ml recombinant human IL-2 (Shanghai Huaxin High Biotechnology). All cells were cultured at 37°C in a humidified atmosphere containing 5% CO_2_ and were routinely tested for mycoplasma contamination.

### Construction of Lentiviral CAR-Expression Vector

**FIGURE 1:**
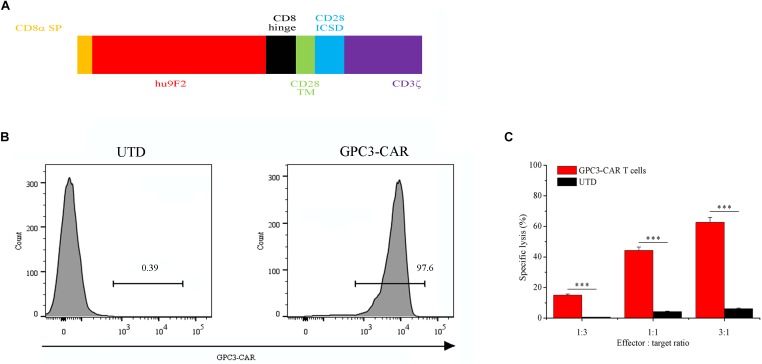
Characterization of second-generation GPC3-specific CAR T cells. **(A)** Schematic representation of second-generation GPC3-specific CAR. CD8α SP, CD8α signal peptide; hu9F2, a humanized GPC3-specific single chain antibody fragment; CD8 hinge, the hinge domain of the CD8α molecule; CD28 TM, the transmembrane region of the human CD28 molecule; CD28 ICSD, the intracellular signaling domain of the human CD28 molecule; CD3ζ, the intracellular signaling domain of CD3 molecule. **(B)** Flow cytometric analysis of the expression of GPC3-specific CAR (GPC3-CAR) on the surface of untransduced (UTD, Left) and transduced (Right) human T cells with lentiviral vector. **(C)** *In vitro* cytotoxic activity of GPC3-CAR T cells against HCC cell PLC/PRF/5 at effector:target ratios of 3:1, 1:1, and 1:3. Data were the mean ± SD from triplicates. Bars, SD. ^∗∗∗^*P* < 0.001 by 2-tailed unpaired *t-*tests.

The lentiviral expression vector (pRRLSIN-hu9F2-28Z) encoding GPC3-specific second-generation CAR was constructed using a pRRLSIN lentiviral vector backbone. The CAR (**Figure 1A**) comprised CD8α signal peptide, a humanized GPC3-specific single chain antibody fragment (scFv, hu9F2) (Bi et al., 2017), the hinge domain of the CD8α molecule (nucleotides 412–546, GenBank NM 001768.6), the transmembrane region (nucleotides 457–537, GenBank NM 006139.3) and the intracellular signaling domain (nucleotides 538–660, GenBank NM 006139.3) of the human CD28 molecule, and the intracellular signaling domain of CD 3ζ molecule (nucleotides 154–492, GenBank NM 198253.2). MluI site and SalI site were added at the 5′ end and the 3′ end of the sequence encoding the CAR, respectively. The DNA fragment encoding the CAR with MluI/SalI sites was synthesized by Genewiz (Suzhou, China), and then, was inserted into the MluI/SalI site of the EF-1α promoter-based lentiviral expression vector pWPT-eGFP (Wang et al., 2011).

### Lentivirus Production

The generation of lentivirus was performed according to the method described by Wu et al. (2017). Briefly, as the confluence reached 95%, HEK-293T cells were transfected with pRRLSIN-hu9F2-28Z and the packaging constructs (RRE/REV, and VSVG) using a polyethylenimine (PEI)-based DNA transfection reagent. Then, the culture medium was replaced with fresh DMEM containing 2% FBS after 6 h of the addition of PEI/DNA complex. After 72 h of transfection, virus was harvested from the conditioned medium and filtered using a 0.45 μm filter unit (Millipore, United States) to remove cell debris. Subsequently, the virus was concentrated and purified with polyethylene glycol.

### Activation, Transduction, and Expansion of Human T Cells

Peripheral blood mononuclear cells were stimulated for 48 h using anti-CD3/anti-CD28 magnetic beads (Invitrogen, United States) at a bead:cell ratio of 1:1. Then, the activated T cells were transduced with lentivirus at a multiplicity of infection (MOI) of 10 on the RetroNectin (Takara, Japan) coated plates. On day 4 post-stimulation, the magnetic beads were removed. The transduced T cells were maintained at a density of 5 × 10^5^ cells/ml, and the recombinant human IL-2 were added to a final concentration of 500 U/ml every other day.

### Design and *in vitro* Transcription of Guide RNA

The gRNA was designed using the CRISPR Design Tool^1^. Considering that simultaneous use of dual gRNAs to target an individual gene can significantly improve the gene-editing efficiency mediated by CRISPR/Cas9 system (Zhou et al., 2014), in this study, two gRNAs were used for the disruption of the PD-1, and both gRNAs targeted to the sequence within exon 1 of the gene *PDCD1* expressing the PD-1. The DNA fragments (**Supplementary Table S1**) containing the T7 promoter, 20 bp targeting sequence, and gRNA scaffold, were synthesized by Genewiz (Suzhou, China), and then used as the template for *in vitro* transcription of both gRNAs using MEGAshortscript^TM^ T7 Transcription Kit (Thermo Fisher Scientific, United States). Two targeting sequences used in this study were listed as following: PD-1-gRNA-1: GTCTGGGCGGTGCTACAACT; and PD-1-gRNA-2: GGCCAGGATGGTTCTTAGGT. The amplification of template for *in vitro* transcription was performed by PCR using the primer pairs Temp-Forward (GTTAATACGACTCACTATA) plus Temp-Reverse (AAAAAAAGCACCGACTCG GTGCCA). The product of *in vitro* transcription was purified using MEGAclear^TM^ Transcription Clean-Up Kit (Thermo Fisher Scientific, United States), and eluted into the nuclease-free water.

### Generation of PD-1 Knockout CAR T Cells

On day 3 post-transduction by lentivirus (i.e., day 5 post-stimulation with anti-CD3/anti-CD28 beads), 3 μg Cas9 protein [New England Biolabs (NEB), United States] was mixed with 3 μg gRNAs, and the mixture was incubated for 10 min at room temperature. Then, the 5 × 10^6^ CAR T cells were electroporated with the CRISPR reagents of Cas9 protein and gRNAs by the Nucleofector 2b Device (Program: T-023) (Lonza, Germany) using Human T Cell Nucleofector^®^ Kit (VPA-1002, Lonza, Germany) according to the procedure described by the manufacturer. Meanwhile, as the control (Cas9 Mock), the 5 × 10^6^ CAR T cells were electroporated only with 3 μg Cas9 protein but without gRNAs.

### Analysis of Allele Modification

The gene editing efficiency and the potential off-target mutations were determined on day 3 post-electroporation. The genomic DNA from tested cells was purified using the QIAamp DNA Mini Kit (Qiagen, United States). The DNA fragment spanning the gene-editing target sites was amplified by PCR from the genomic DNA using the primer pairs of *PDCD1*-detect-Forward (CAAGGAGATAAGCAAGCCATTT) plus *PDCD1*-detect-Reverse (AAGCCAAGGTTAGTCCCACAT). The DNA fragments spanning the potential off-target sites were amplified by PCR from the genomic DNA using the primer pairs listed in the **Supplementary Table S2**.

(1) Sequencing and TIDE analysis: The allele modification frequencies were quantified by clonal sequencing analysis and TIDE analysis of PCR amplicon spanning the gene-editing target sites. The purified DNA fragments spanning the gene-editing target sites were ligated into the pMD-20T vector (Takara, Japan), and a total of 60 colonies were selected for DNA sequencing (Genewiz, Suzhou, China). As for the evaluation of Tracking of Indels by Decomposition (TIDE) (Brinkman et al., 2014), the purified DNA fragments spanning the gene-editing target sites were Sanger-sequenced using the primers PD-1-seq-Forward (5′TCCCCAGCACTGCCTCTGTCACTC3′) and PD-1-seq-Reverse (5′CACAGCTC AGGGTAAGGGGCAGA3′) by Genewiz (Suzhou, China), and the analysis of each sequence chromatogram was carried out using the online TIDE software available at http://tide.nki.nl. The sequence from a Cas9 mock-transfected sample was used as the reference sequence. Parameters were set to the maximum indel size of 50 nucleotides and the decomposition window to cover the largest possible window with high quality traces. When the TIDE analysis was below the detection sensitivity of 1.5%, it was set to 0%. All the sequencing primers which were used for TIDE off-target analysis were listed in **Supplementary Table S3**.

(2) T7EN1 assay: The mismatched DNA can be detected by the T7EN1 assay (Niu et al., 2014). After purification, the 200 ng of DNA fragment spanning the gene-editing target sites was denatured and reannealed in 1× NEBuffer 2 (NEB, United States) in a thermocycler with the following steps (Guschin et al., 2010): 95°C, 5 min; 95–85°C at -2°C/s; 85–25°C at -0.1°C/s; hold at 4°C. Subsequently, 10 U of T7 Endonuclease I (T7EN1) (NEB, United States) were added into the hybridized DNA fragments and reaction mixtures were incubated for 15 min at 37°C. Following digestion, 1 μl of proteinase K was added and incubated for 5 min at 37°C to inactivate the enzyme and stop the reaction. The DNA fragments digested by T7EN1 enzyme were separated by 1% agarose gel electrophoresis, stained with ethidium bromide.

### *In vitro* Cytotoxicity Assays

The *in vitro* cytotoxicity was evaluated by the lactate dehydrogenase (LDH) release assay with CytoTox96 Non-Radioactive Cytotoxicity Kit (Promega, United States), and the assay was performed according to the manufacturer’s instructions. Briefly, 1 × 10^4^ HCC cells (target cells) were co-cultured with the genetically modified (or not) T cells (effector cells) at an indicated effector:target ratio in a total volume of 100 μL in the wells of 96-well V-bottom plates for 18 h at 37°C. The RPMI 1640 medium (Gibco, United States) containing 10% FBS was used in the co-cultures. Then, the supernatants were collected by centrifugation at 250 ×*g* for 4 min at room temperature, and the released LDH in the supernatants was measured using colorimetric method at 490 nm. The spontaneous release of LDH from target and effector cells and the maximum release of LDH from target cells were determined in parallel. The percentage of specific cell lysis was calculated based on the following formula:

$$\begin{array}{r} 100\times (\text{experimental release}-\text{target spontaneous release}-\\ \text{effector spontaneous release})/(\text{target maximal release}-\\ \text{target spontaneous release}) \end{array}$$

### Cytokine Release Assay

Firstly, 1 × 10^4^ HCC cells (target cells) were co-cultured with the genetically modified (or not) T cells (effector cells) at an effector:target ratio of 1:1 in a total volume of 100 μL in the wells of 96-well V-bottom plates for 18 h at 37°C. The RPMI 1640 medium (Gibco, United States) containing 10% FBS was used in the co-cultures. Then, the supernatant was collected by centrifugation at 250 ×*g* for 4 min at room temperature. The concentrations of IFN-gamma and IL-2 in the supernatant were measured by enzyme-linked immunosorbent assay (ELISA) using the Human IFN-gamma ELISA kit (EK1802) and Human IL-2 ELISA kit (EK1022) (both from Multisciences Biotech, Hangzhou, China) according to the manufacturer’s instructions. As for the mouse blood, after it was collected and clotted at 4°C, and then, the serum was used for the detection of cytokine as above.

### Flow Cytometry

For all experiments [except for intracellular Akt and phospho-Akt (Ser^473^) staining], the cells were analyzed by surface antibody staining. The following antibodies with indicated specificity and the appropriate isotype controls were used: anti-human CD3-FITC (11-0036-41), anti-human CD8-FITC (11-0086-42), anti-human CD25-PE (12-0259-41), anti-human PD-L1-PE (12-5983-42), mouse IgG_1_-FITC isotype control (11-4714-81), and mouse IgG_2a_-FITC isotype control (11-4724-42) (all from Thermo Fisher Scientific, United States); anti-human CD4-FITC (555346), anti-human CD4-PE (555347), anti-human PD-1-BV421 (564323), mouse IgG_1_-PE isotype control (555749), and mouse IgG_1_-BV421 isotype control (562438) (all from BD Biosciences, United States); anti-human CD69-PerCP (310928) and Mouse IgG1-PerCP (400148) (both from BioLegend, United States). The CAR expression was evaluated by the biotinylated goat anti-human Fab antibody (109-066-006, Jackson ImmunoResearch, United States), followed by PE-conjugated streptavidin (12-4317-87, eBioscience, United States) staining, if not specifically indicated. For the intracellular Akt and phospho-Akt (Ser^473^) analysis, CAR T cells were first stained by the biotinylated goat anti-human Fab antibody and FITC-conjugated streptavidin (11-4317-87, eBioscience, United States) on ice after the CAR T cells harvested from the 48-h coculture of GPC3-CAR T and PLC/PRF/5 cells at a ratio of 1:1, and then, the cells were fixed, permeabilized, and stained using the antibodies of an anti-Akt mouse mAb (2920S), an anti-phospho-Akt (Ser^473^) rabbit mAb (4060S), a mouse mAb IgG1 isotype control (5415S) and Rabbit mAb IgG isotype control (3900S) (all from Cell Signaling Technology, United States) according to the manufacturer’s protocol, followed by PE-conjugated secondary antibodies of anti-mouse IgG (8887S, Cell Signaling Technology, United States) and anti-rabbit IgG (8885S, Cell Signaling Technology, United States). Fixable, viable stain 780 (565388, BD Biosciences, United States) was used for discriminating live from dead cells according to the manufacturer’s instruction. Flow cytometric measurements were carried out using a FACSCelesta^TM^ flow cytometer (BD Biosciences, United States) equipped with FACSDiva software for data acquisition. FlowJo software (Tree Star, United States) was used for data analysis.

### Mouse Xenograft Model

Six- to eight-week-old female NSG mice were housed and treated at the Experimental Animal Center of Shanghai Jiao Tong University School of Medicine (Shanghai, China) in specific pathogen-free conditions. The animal experiments were performed in accordance with the guidelines and regulations approved by the Shanghai Medical Experimental Animal Care Commission. Subcutaneous xenograft tumors were established by injection of 3 × 10^6^ PLC/PRF/5 in PBS. When the tumor volume reached 100–200 mm^3^, mice bearing the tumor were randomly allocated into four groups (*n* = 7) and assigned to receive one of the following intravenous injections: (1) sterile PBS, (2) 5 × 10^6^ UTD in sterile PBS, (3) 5 × 10^6^ wild-type CAR T cells in sterile PBS, and (4) 5 × 10^6^ PD-1-deficient CAR T cells in sterile PBS. Tumor burden was measured by an electronic caliper, and tumor volume was calculated based on the following formula as described by Gao et al. (2014):_V = L × W × W / 2_, where L was length and W was width. When the mean tumor volume in the control group reached 1,500–2,000 mm^3^, mice were euthanized.

### Quantitative Real-Time PCR

mRNA was isolated from cells using TRIzol reagent (15596026, Thermo Fisher Scientific, United States) and then reverse transcribed into cDNA using the GoScript^TM^ Reverse Transcription system (A5001, Promega, United States) according to the manufacturer’s instructions. All the quantitative real-time PCR reactions were performed with TB Green^TM^ premix Ex Taq^TM^ II (Tli RNaseH Plus) (RR820A, Takara, Japan) according to the manufacturer’s protocol on an ABI 7500 RT-PCR system (Applied Biosystems, United States), using the primers in the **Supplementary Table S4**. Glyceraldehyde 3-phosphate dehydrogenase was used as the internal control. The relative quantification was calculated by the 2^-ΔΔCt^ method (Livak and Schmittgen, 2001).

### Immunohistochemistry

To assess the infiltration of adoptive T cells in the xenografts after treatment, the tumor tissues were fixed with formalin, embedded in paraffin, and serially sectioned at 2-μm thickness. The sections of fixed and embedded tumor tissues were immunostained with an anti-CD3e monoclonal antibody (MA5-14524, Thermo Fisher Scientific, United States) at a 1:150 dilution. Images were taken under a Leica SCN400 system (Leica Microsystems, Germany) at 20× magnification.

### Statistics

All data were shown as mean ± standard deviation (SD). Two-tailed unpaired *t*-tests, one-way ANOVA with Turkey *post hoc* tests, correlation and regression analysis were carried out using GraphPad Prism version 6.0 (GraphPad Software Inc., United States). ^∗^*P* < 0.05, ^∗∗^*P* < 0.01, and ^∗∗∗^*P* < 0.001 were considered statistically significant.

## Results

### Generation of GPC3-Specific CAR T Cells, and Cytotoxicity of the CAR T Cells Against HCC Cell PLC/PRF/5

As shown in **Figure 1A**, GPC3-specific second-generation CAR comprised CD8α signal peptide, a humanized GPC3-specific single chain antibody fragment (scFv, hu9F2) (Bi et al., 2017), the hinge domain of the CD8α molecule, the transmembrane region and the intracellular signaling domain of the human CD28 molecule, and the intracellular signaling domain of CD 3ζ molecule. GPC3-CAR T cells were generated by lentiviral vector transduction as described in the “Materials and Methods” section. The expression of CAR was evaluated by flow cytometry on day 3 post-transfection. As shown in **Figure 1B**, the percentage of the CAR-positive T cells reached 97.6%, indicating that the efficiency of lentiviral transduction was high, and GPC3-CAR T cells were successfully generated. Furthermore, as shown in **Figure 1C**, it was found that GPC3-CAR T cells showed the significantly (*P* < 0.001) stronger cytotoxicity on HCC cell PLC/PRF/5 compared with the UTD, and the cytotoxicity was enhanced with the increase of effector:target ratio from 1:3 to 3:1, indicating that the cytotoxicity of GPC3-CAR T cells was dose-dependent.

### Remarkable Upregulation of PD-L1 Expression on PLC/PRF/5 After Encountering GPC3-CAR T Cells

**FIGURE 2:**
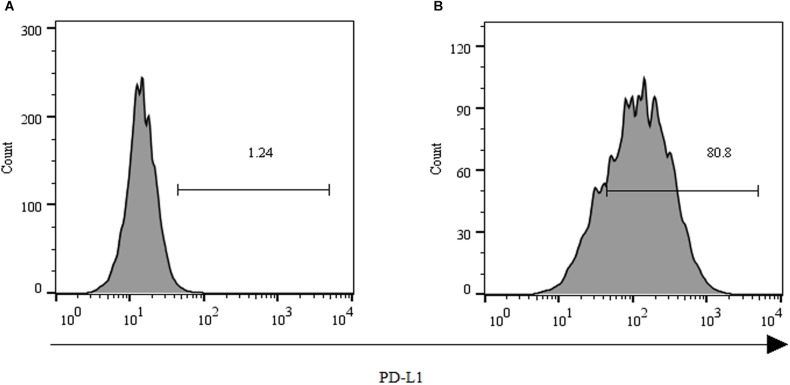
Expression of PD-L1 on human HCC PLC/PRF/5 cells. **(A)** Human HCC PLC/PRF/5 cells were cultured alone in the absence of GPC3-CAR T cells in RPMI 1640 medium containing 10% FBS. **(B)** Human HCC PLC/PRF/5 cells were cocultured with GPC3-CAR T cells at an effector:Target ratio of 1:1 for 18 h in RPMI 1640 medium containing 10% FBS. PD-L1 was determined by flow cytometry in the CD3-negative gate, and the fixable, viable stain 780 was used for discriminating live from dead cells.

As shown in **Figure 2**, over 80% of the PLC/PRF/5 cells expressed the inhibitory ligand PD-L1 after coculture with GPC3-CAR T cells at an effector:target ratio of 1:1 for 18 h. However, only 1.24% of the PLC/PRF/5 cells were PD-L1-positive, when the PLC/PRF/5 cells were normally cultured in the absence of GPC3-CAR T cells. These results indicated that the expression of PD-L1 on HCC PLC/PRF/5 is inducible, and the expression can be up-regulated after PLC/PRF/5 encountering GPC3-CAR T cells.

### Preparation and Characterization of PD-1-Deficient GPC3-Specific CAR T Cells

**FIGURE 3:**
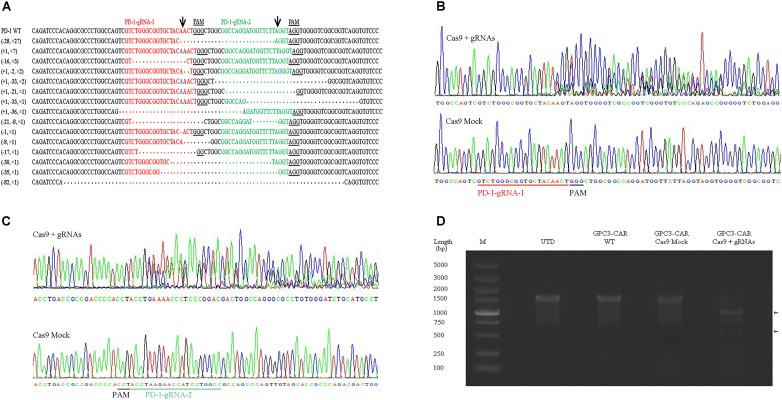
CRISPR/Cas9 efficiently disrupted the gene expressing PD-1 in GPC3-CAR T cells. **(A)** Indels observed by clonal sequence analysis of PCR amplicons from the CRISPR-edited region in the gene expressing PD-1. Blue base or dot in the clonal sequences indicated insertion or deletion base, respectively. The number prefixed a “+” or “–” character in the bracket before a clonal sequence indicated the number of insertions or deletions in the corresponding clonal sequence, respectively. The number prefixed with a “×” character in the bracket before a clonal sequence indicated the number of the corresponding indels profile in the sixty clonal amplicons. Arrows indicated the putative cleavage sites. **(B,C)** The chromatograms from the Sanger sequencing of the PCR amplicon spanning the PD-1 CRISPR gRNAs [PD-1-gRNA-1 **(B)** and PD-1-gRNA-2 **(C)]** target sites within the exon 1 of the gene expressing PD-1. **(D)** Detection of the CRISPR-mediated disruption of PD-1 by a mismatch-selective T7EN1 nuclease assay on the DNA (spanning the gRNAs target sites) amplified from the genomic DNA of the cells shown.

**FIGURE 4:**
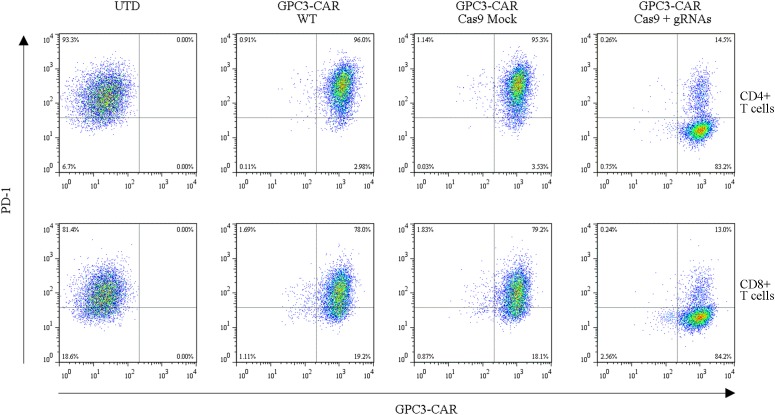
Efficient disruption of PD-1 expression on the surface of GPC3-CAR T cells. PD-1 and CAR expression on the surface of T cells were detected by flow cytometry on day 3 after the re-stimulation with anti-CD3/anti-CD28 beads. UTD, untransduced T cells; WT, wild type.

To further investigate the effect of PD-1-mediated immunosuppressive pathway on the efficacy of GPC3-CAR T cells against HCC, the PD-1-deficient GPC3-CAR T cells was generated through direct delivery of CRISPR/Cas9 gene-editing system into the CAR T cells by electroporation on day 3 post-lentiviral transduction. Gene-editing efficiency was evaluated by sequencing and T7 endonuclease I (T7EN1)-based mutation detection assay, 2–4 days after nucleofection. Clonal sequencing indicated the genomic editing efficiency reached 85%. There were fifteen kinds of indels resulted from the non-homologous end joining (NHEJ) repair in 60 sequenced clones (**Figure 3A**), and deletion mutations were the most prominent among the observed Indels. Multiple peaks flanking the PD-1 target site appeared in the Sanger sequencing data of the PCR amplicon spanning the gene-editing target sites (**Figures 3B,C**), which confirmed that the shift of genomic reading frame occurred downstream of the target sites. The TIDE analyses showed that the indels frequencies reached 77.9 and 76.8% at the target sites of PD-1-gRNA-1 and PD-1-gRNA-2, respectively. In the T7EN1-based mutation detection assay (**Figure 3D**), the obvious cleavage further confirmed the mutation at the genomic locus of PD-1. Furthermore, the expression of PD-1 was characterized by flow cytometry on day 3 post-restimulation of CRISPR-edited CAR T cells with anti-CD3/anti-CD28 beads. As shown in **Figure 4**, above 83% reductions of CAR+ PD-1+ cells were observed in both CD4- and CD8-gated cells, indicating that PD-1 was successfully disrupted with high efficiency in both CD4-positive and CD8-positive GPC3-CAR T cells. In addition, the top five potential off-target sites for each gRNA in the CRISPR-edited GPC3-CAR T cells were sequenced, and no mutation was found at any of these sites using TIDE analysis (**Supplementary Table S5**). Taken together, PD-1-deficient GPC3-specific CAR T cells were successfully and efficiently generated using the CRISPR/Cas9 gene-editing system.

Given that the surface expression of PD-1 on CAR T cell with intact genomic DNA was low (PD-1-positive cell percentage: 1.18% on day 9 post the activation of primary T cells, **Supplementary Figure S1**) after expansion if without the restimulation by anti-CD3/anti-CD28 beads, and moreover, repeated stimulation can cause T cells exhaustion (Cherkassky et al., 2016), it was difficult to enrich the PD-1 deficient CAR T cells. Therefore, the generated PD-1 deficient CAR T cells used for the following *in vitro* and *in vivo* assays were a mosaic population of cells with the disrupted or intact PD-1, although the GPC3-CAR T cells with the disrupted PD-1 were the prominent population.

### Disruption of PD-1 in GPC3-CAR T Cells Enhanced the Specific CAR-Dependent Cytotoxic Function and Cytokines Production *in vitro*, and Did Not Affect Subsets Constitution and Activation Status of the CAR T Cells

**FIGURE 5:**
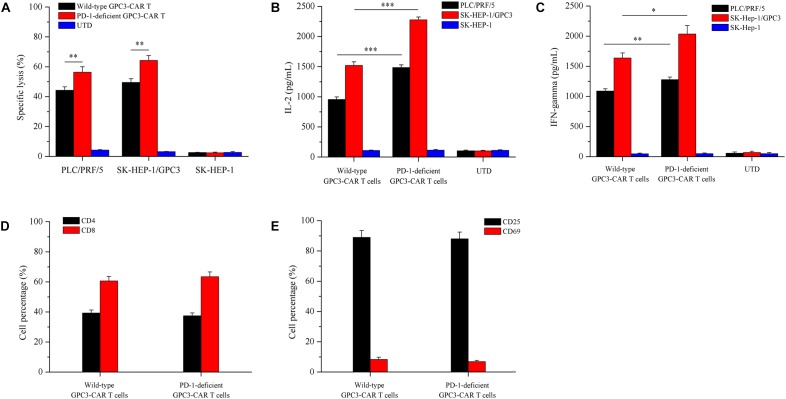
Effects of disruption of PD-1 on cytotoxicity, cytokines production, and phenotype of GPC3-CAR T cells *in vitro*. **(A)** Cytotoxic activities of UTD and GPC3-CAR T cells with intact or deficient PD-1 against GPC3-positive (PLC/PRF/5 and SK-HEP-1/GPC3) and GPC3-negative (SK-HEP-1) HCC cells at an effector:target ratio of 1:1. **(B,C)** The production of IL-2 **(B)** and IFN-gamma **(C)** by the UTD and GPC3-CAR T cells with intact or deficient PD-1 cocultured with GPC3-positive (PLC/PRF/5 and SK-HEP-1/GPC3) or GPC3-negative (SK-HEP-1) HCC cells at an effector:target ratio of 1:1. **(D)** CD4 and CD8 subsets constitution of wild-type and PD-1-deficient GPC3-CAR T cells. The expressions of CD4 and CD8 on CAR T cells were measured by flow cytometry. **(E)** The expressions of early (CD69), and intermediate or late (CD25) activation markers on cell surface of wild-type and PD-1-deficient GPC3-CAR T cells. The expressions of CD25 and CD69 were determined by flow cytometry. Data shown were mean ± SD from triplicates. Bars, SD. ^∗^*P* < 0.05, ^∗∗^*P* < 0.01, and ^∗∗∗^*P* < 0.001 by one way ANOVA with Turkey *post hoc* test.

To evaluate whether the disruption of PD-1 affected specific CAR-dependent cytotoxic function and cytokines secretion of GPC3-CAR T cells, the *in vitro* tumor-lysis activity and secreted cytokines of the CRISPR-edited (or not) CAR T cells were investigated by the coculture of CAR T cells and each of various GPC3-positive (PLC/PRF/5 and SK-HEP-1/GPC3) or GPC3-negative (SK-HEP-1) HCC cells. As shown in **Figure 5A**, the PD-1 deficient GPC3-CAR T cells showed significantly (*P* < 0.01) stronger tumor-lysis activity against GPC3-positive PLC/PRF/5 and SK-HEP-1/GPC3 HCC cells compared with wild-type GPC3-CAR T cells, and the anti-tumor activities of PD-1 deficient GPC3-CAR T cells against PLC/PRF/5 and SK-HEP-1/GPC3 HCC cells were 1.25 and 1.30 times higher than those of wild-type GPC3-CAR T cells, respectively, indicating that the disruption of PD-1 enhanced the cytotoxic activity of GPC3-CAR T cells. Meanwhile, the anti-tumor activity of PD-1 deficient GPC3-CAR T cells against GPC3-negative SK-HEP-1 HCC cells was limited (<5%) and similar to that of UTD and wild-type GPC3-CAR T cells, indicating that the disruption of PD-1 did not affected the cytotoxic specificity of GPC3-CAR T cells. As shown in **Figures 5B,C**, the concentrations of IL-2 and IFN-gamma in the cocultures of PD-1 deficient GPC3-CAR T cells and GPC3-positive HCC cells (PLC/PRF/5 and SK-HEP-1/GPC3) was significantly higher than those in the coculture of wild-type GPC3-CAR T cells and GPC3-positive HCC cells, but PD-1 deficient GPC3-CAR T cells similar to UTD and wild-type GPC3-CAR T cells produced little or even negligible cytokines in the coculture with GPC3-negative SK-HEP-1, indicating that cytokines production by the GPC3-CAR T was CAR-dependent and enhanced by the disruption of PD-1. In addition, as shown in **Figures 5D,E**, no significantly statistical difference was found between PD-1-deficient and wild-type GPC3-CAR T cells in the CD4-positive, CD8-positive, CD69 (early activation marker)-positive or CD25 (intermediate or late activation marker)-positive cell percentage, indicating that CD4 and CD8 subsets constitution and activation status of GPC3-CAR T cells were stable with the disruption of endogenous PD-1. Taken together, the disruption of PD-1 in GPC3-CAR T cells enhanced specific CAR-dependent cytotoxic function and cytokines secretion, and did not affect the CD4 and CD8 subsets constitution and activation status of the GPC3-CAR T cells.

### Disruption of PD-1 Increased the Levels of Akt Activation and Bcl-xL Expression in the GPC3-CAR T Cells After Combating the HCC Cells

**FIGURE 6:**
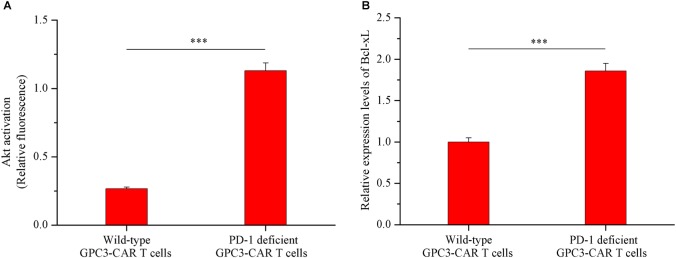
Akt activation and Bcl-xL expression in GPC3-CAR T cells with deficient or intact PD-1. The GPC3-CAR T cells were isolated from the 48-h coculture with PLC/PRF/5. **(A)** Akt activation in GPC3-CAR T cells with deficient or intact PD-1 was cytometrically measured as the ratio of phospho-Akt (Ser^473^)/Akt in an intracellular stain. Phospho-Akt (Ser^473^) and Akt were determined by flow cytometry in the CAR-positive gate, and the fixable, viable stain 780 was used for discriminating live from dead cells. **(B)** mRNA expression levels of Bcl-xL in GPC3-CAR T cells with deficient or intact PD-1 determined by quantitative real-time PCR. Data were the mean ± SD from triplicates. Bars, SD. ^∗∗∗^*P* < 0.001 by 2-tailed unpaired *t*-tests.

As shown in **Figures 6A,B**, both the Akt activation status and the expression of anti-apoptotic protein Bcl-xL in PD-1 deficient GPC3-CAR T cells was significant (*P* < 0.001) increased compared with that in the wild-type GPC3-CAR T cells after 48 h of coculture with native PD-L1-expressing GPC3-positive PLC/PRF/5 HCC cells. The ratio of phospho-Akt/Akt in the PD-1 deficient GPC3-CAR T cells was 4.35 times higher than that in the wild-type GPC3-CAR T cells, and meanwhile, the expression level of Bcl-xL in the PD-1 deficient GPC3-CAR T cells was 1.86 times higher than that in the wild-type GPC3-CAR T cells after 48 h of coculture with native PD-L1-expressing GPC3-positive PLC/PRF/5 HCC cells. Taken together, the disruption of PD-1 increased the levels of Akt activation and anti-apoptotic protein Bcl-xL expression after combating the HCC cells.

### Disruption of PD-1 Enhanced *in vivo* Antitumor Efficacy, Survival, Cytokines Production, and Infiltration of GPC3-CAR T Cells

**FIGURE 7:**
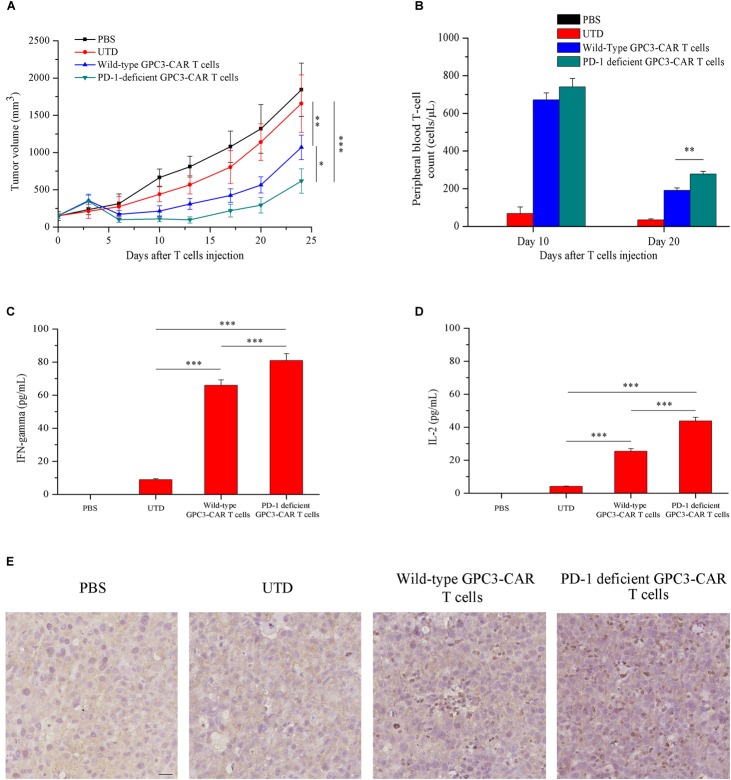
*In vivo* antitumor activities, persistence, cytokines production and infiltration of GPC3-CAR T cells with intact or deficient PD-1 in the established subcutaneous HCC tumor xenograft model with PLC/PRF/5. **(A)** Growth curve of PLC/PRF/5 xenografts treated with the indicated T cells or PBS (*n* = 7). At the endpoint, the residual tumors treated with PD-1-deficient GPC3-CAR T cells were significantly (^∗^*P* < 0.05) smaller than those treated with wild-type GPC3-CAR T cells. **(B)** The quantities of circulating human T cells from the mice bearing PLC/PRF/5 xenografts treated with the indicated T cells or PBS on days 10 and 20 after T cells or PBS injection. The quantitative analysis was completed using TruCount tubes. On day 20 after T cells or PBS injection, PD-1-deficient GPC3-CAR T cells showed the significantly (^∗∗^*P* < 0.01) enhanced *in vivo* persistence compared with wild-type GPC3-CAR T cells. **(C,D)** The levels of IFN-gamma **(C)** and IL-2 **(D)** in mouse serum evaluated by ELISA at the endpoint of the experiment. Data shown were mean ± SD from each treatment group. Bars, SD. ^∗^*P* < 0.05, ^∗∗^*P* < 0.01, and ^∗∗∗^*P* < 0.001 by one way ANOVA with Turkey *post hoc* test. **(E)** Infiltration of human T cells in the tumor tissues treated with indicated genetically engineered (or not) T cells. Formalin-fixed, paraffin-embedded tumor sections were consecutively cut, and then, stained for human CD3e to detect the human T cell infiltration (brown). Scale bar, 50 μm.

Given that HCC cell PLC/PRF/5 natively expressed GPC3 on the cell surface in contrast to SK-HEP-1/GPC3, the efficacy of PD-1-deficient GPC3-CAR T cells was evaluated *in vivo* in NSG mice bearing established PLC/PRF/5 subcutaneous xenograft tumors. As shown in **Figure 7A**, tumor growth was significantly (*P* < 0.01) inhibited in mice treated with GPC3-CAR T cells compared with those treated with UTD or PBS. Moreover, PD-1-deficient GPC3-CAR T cells showed stronger anti-tumor activity compared with the wild-type GPC3-CAR T cells. At the endpoint of the animal experiment, the tumor volumes in the mice treated with PD-1-deficient GPC3-CAR T cells were significantly (*P* < 0.05) smaller than those treated with wild-type GPC3-CAR T cells, and tumor weights in the mice treated by the PD-1-deficient GPC3-CAR T cells were significantly (*P* < 0.01) lighter than those in other groups (**Supplementary Figure S2**), indicating that the disruption of PD-1 enhanced the anti-tumor activity of GPC3-CAR T cells.

Meanwhile, to investigate the effect of the disruption of PD-1 on the *in vivo* survival of GPC3-CAR T cells, the density of GPC3-CAR T cells in mouse peripheral blood was tested. It was found that, as shown in **Figure 7B**, while the survivals of both wild-type GPC3-CAR T and PD-1-deficient GPC3-CAR T cells in mice decreased with time, the density of PD-1-deficient GPC3-CAR T cells was significantly (*P* < 0.01) higher than that of wild-type GPC3-CAR T cells on day 20 post-CAR T cells infusion. The results suggested that the disruption of PD-1 benefited the *in vivo* survival of GPC3-CAR T cells. In addition, correlation analyses showed that the density of GPC3-CAR T cells in mouse peripheral blood significantly (*P* < 0.05) negatively correlated with the tumor burdens in both treatment groups of wild-type and PD-1-deficient GPC3-CAR T cells. Furthermore, the levels of IFN-gamma and IL-2 in the mouse blood of the group treated by the PD-1-deficient GPC3-CAR T cells were significantly higher than the counterparts in those treated by wild-type GPC3-CAR T cells as shown in **Figures 7C,D**. The immmunochemical analysis (**Figure 7E**) showed that there were more T cells infiltration in the tumor tissues treated by PD-1-deficient GPC3-CAR T cells compared with those treated by wild-type GPC3-CAR T cells, indicating that the disruption of PD-1 enhanced the infiltration of GPC3-CAR T cells in tumor tissues.

### Disruption of PD-1 Enhanced Inhibition of Tumor-Relate Genes Expression in Xenografts Caused by the GPC3-CAR T Cells

**FIGURE 8:**
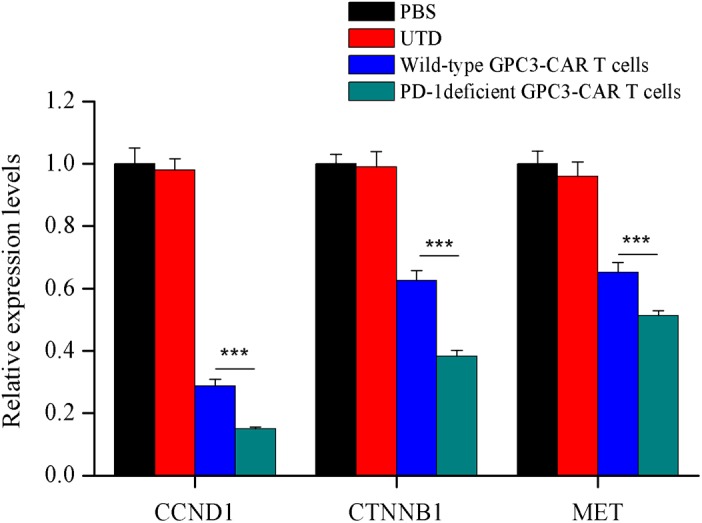
Characterization of the mRNA expression levels of tumor related genes in xenografts treated with various genetically engineered human T cells. The mRNA expression levels of tumor related genes were evaluated by quantitative real-time PCR. CCND1, cyclin D1; CTNNB1, catenin beta 1; MET, MET proto-oncogene, receptor tyrosine kinase. Data were the mean ± SD from triplicates. Bars, SD. ^∗∗∗^*P* < 0.001 by one way ANOVA with Turkey *post hoc* test.

In order to investigate the effect of PD-1 deficient GPC3-CAR T cells on the tumor-related genes expression in xenograft tumors established with PLC/PRF/5, quantitative reverse transcription PCR was carried out to characterize the mRNA expression levels of tumor-related genes of *CCND1* (cyclin D1), *CTNNB1* (catenin beta-1) and *MET* (MET proto-oncogene, receptor tyrosine kinase) in xenografts treated with various genetically engineered (or not) T cells or PBS. As shown in the **Figure 8**, both wild-type GPC3-CAR T cells and those with deficient PD-1 significantly (*P* < 0.001) inhibited the expression of the three tumor-related genes in xenografts, and the PD-1 deficient GPC3-CAR T cells caused the inhibition at a significantly (*P* < 0.001) larger degree compared with wild-type GPC3-CAR T cells. Taken together, disruption of PD-1 enhanced the inhibition of tumor-relate genes expression in xenografts caused by the GPC3-CAR T cells.

## Discussion

Hepatocellular carcinoma is a prevalent cancer worldwide with one of the worst prognoses, and the curative treatment option is only for the patients with limited tumor burden (Yoshiji et al., 1998; Callegari et al., 2013; Yu et al., 2018). HCC is a uniquely immunosuppressive cancer (Obeid et al., 2018). Immunosuppressive intrahepatic environment, which restricts antitumor immunity and promotes tumor progression, is a significant obstacle to treatment of liver cancer (Knolle and Thimme, 2014; Thorn et al., 2016). The majority of liver myeloid-derived suppressor cells were found to express immune-inhibitory ligand PD-L1 (Thorn et al., 2016). Chen et al. (2016) found PD-L1 expression in the primary human HCC surgical specimens. In current study, the upregulation of PD-L1 expression was observed on the HCC cell PLC/PRF/5 exposed to the GPC3-CAR T cells. In this sense, the efficacy of CAR T cell therapy could be more prone to be challenged by the inhibitory PD-1/PD-L1 pathway in the immunosuppressive HCC microenvironment. In the present study, CRISPR-mediated disruption of PD-1 led to enhanced antitumor activity against HCC. Although the previous studies have showed that, in some tumor models, the disruption of PD-1 enhanced the antitumor activity of CAR T cells, those studies mainly focused on the leukemia and pancreatic cancer cells, and most of tumor models in those studies were not derived from the native PD-L1-expressing tumor cells (Ren et al., 2017; Rupp et al., 2017). The functions of CAR T cells could be differential among those with the distinct co-stimulatory domains (Carpenito et al., 2009; Guedan et al., 2014, 2018; Zhao et al., 2015), and all the co-stimulatory domains in the abovementioned previous studies were 4-1BB, which was different from CD28 employed in the current study. To our best knowledge, the current study combined 28ζ CAR modification and the CRISPR-mediated disruption of endogenous inhibitory immune checkpoint receptor PD-1 in adoptive T cell immunotherapy of native PD-L1-expressing HCC for the first time.

Robust expansion and persistence of CAR T cells are critical for the *in vivo* antitumor efficacy (Louis et al., 2011; Maude et al., 2014; Guedan et al., 2018). Menger et al. (2016) showed that TALEN-mediated inactivation of PD-1 in tumor-reactive lymphocytes promoted T-cell persistence and improved the antitumor efficacy against melanoma and fibrosarcoma *in vivo*, while the CAR was not introduced into the T cells. Cherkassky et al. (2016) demonstrated that cotransduction of PD-1 dominant negative receptor increased the proliferative ability of 28ζCAR T cells and rescued CAR T cells from PD-1 ligand-mediated inhibition. In the current study, the persistence of GPC3-CAR T cells significantly (*P* < 0.05) negatively correlated with the tumor burdens in both treatment groups of wild-type and PD-1-deficient GPC3-CAR T cells. Moreover, CRISPR-mediated disruption of endogenous PD-1 significantly (*P* < 0.01) improved the persistence of 28ζ CAR T cells redirected toward GPC3 *in vivo* as well, associating with the enhanced *in vivo* antitumor efficacy against native PD-L1-expressing HCC.

Repeated antigen stimulation can induce T cell exhaustion and deletion, and human CAR T cells are subject to inhibition of their cytolytic functions upon repeated antigen encounter *in vivo* (Cherkassky et al., 2016). Gargett et al. (2016) showed that GD2-specific CAR T cells underwent potent activation and deletion following antigen encounter, although the activation-induced cell death was reduced by PD-1 blockade. In the current study, although the CRISPR-mediated disruption of endogenous PD-1 benefited the persistence of CAR T cells, the PD-1-deficient CAR T cells were still to decrease *in vivo* with time, which should be related to the exhaustion and deletion of CAR T cells caused by the continual tumor challenge. This could be an important reason for the phenomenon that tumor stopped regression and re-grew after 13 days of the infusion of CAR T cell with deficient PD-1. Additionally, for this phenomenon, it cannot be excluded that inhibition of cytolytic function of PD-1-deficient CAR T cells caused by the compensatory upregulation of alternative checkpoints, considering that the blockade of one checkpoint pathway is often followed by the compensatory upregulation of other alternative immune checkpoint pathways. Koyama et al. (2016) showed that adaptive resistance to therapeutic PD-1 blockade is associated with upregulation of alternative immune checkpoints on the PD-1 antibody bound T cells in lung adenocarcinoma, notably T-cell immunoglobulin mucin-3. Huang et al. (2017) found that blockade of PD-1, LAG-3, or CTLA-4 alone conferred a compensatory upregulation of the other checkpoints on T cells in metastatic ovarian cancer. Henceforth, it will be very important to investigate the compensatory immunosuppressive checkpoints of PD-1: PD-L1/L2 pathway on 28ζ CAR T cells in the HCC microenvironment, and the CRISPR-mediated combinatorial disruption of checkpoints will be beneficial for the 28ζ CAR T cells achieving the sustained regression and eradication of HCC.

Under physiological conditions, the PD-1:PD-L1/L2 pathway prevents excessive effector activities by T cells and promotes the tolerance to self-antigens to avoid the development of autoimmunity (Papaioannou et al., 2016). Although monoclonal antibodies blocking PD-1, such as pembrolizumab and nivolumab, can retrieve the functionality of exhausted T cells and produce potent antitumor immune response in patients with various cancers, the systemic administration of the immune checkpoint pathway blocking antibodies still runs the risk of disrupting immunologic homeostasis, producing unique immune-related adverse effects, and even threatening the life (Gettinger et al., 2015; Larkin et al., 2015; Robert et al., 2015; Weber et al., 2015). The disruption of intrinsic immune checkpoints in T cells through gene editing is considered to be a relatively safer strategy compared with the systemic administration of blocking antibody (Lloyd et al., 2013; June et al., 2015). Su et al. (2016) found the disruption of PD-1 did not change the activation status of human primary T cells not carrying the CAR, while the CAR was not introduced into the T cells. In the current study, the disruption of endogenous PD-1 did not affect the activation status and cytotoxic specificity of CAR T cells, and the cytotoxic function of the PD-1-deficient CAR T cells was still CAR-dependent. However, given that CAR T cells with individual disruption of PD-1 are still likely to express auto-reactive TCRs, there might be the potential autoimmune adverse effects resulted from the PD-1-deficient CAR T cells with intact TCR (Rupp et al., 2017). Therefore, it will be crucial to disrupt TCR for the safe and efficient utilization of the GPC3-CAR T cell with deficient immunosuppressive checkpoint molecules on anti-HCC therapy. Besides, considering the NSG animal model used in this study with severe deficient immune system was largely different from the clinical conditions, henceforth, safe estimation is needed in the immunocompetent animal models before proceeding to clinic.

Additionally, tumor-associated antigens often express at low levels in normal tissues (Simpson et al., 2005; Johnson et al., 2009). GPC3 is expressed in most (72%) of HCC and not in normal liver tissue, but its expression in other normal tissues could not be completely eliminated (Capurro et al., 2003; Baumhoer et al., 2008; Hass et al., 2015). Thus, the on-target, off-tumor toxicity of the GPC3-CAR T cell might occur, even if without the disruption of the PD-1. Chen et al. (2017) found that dual-targeted CAR-T cells co-expressing complementary GPC3 and asialoglycoprotein receptor 1 (a liver tissue-specific protein)-targeted CARs showed relatively potent anti-tumor activity against HCC tumor xenografts with double antigens, but exhibited the restricted antitumor activity against HCC xenografts with a single antigen, indicating that dual-targeted CAR-T cells could be a promising strategy for reducing or avoiding the potential off-tumor toxicities of the GPC3-CAR T cell therapy on HCC. In the present study, although the antitumor activity of GPC3-CAR T cell with deficient PD-1 was CAR-dependent, its off-tumor toxicity cannot be excluded in clinical therapy. Henceforth, combination of the dual-targeted CAR modification and the simultaneous disruption of the TCR and compensatory immunosuppressive checkpoint molecules in T cells will be important for the generation of the highly potent and safe genetically engineered CAR T cells in the therapy of HCC.

A key signaling target of PD-1-mediated inhibition is the PI3K-Akt pathway (Boussiotis, 2016). The previous studies found that the triggering of PD-1-mediated signals blocked the CD28-mediated activation of PI3K and Akt, and the expression of anti-apoptotic protein Bcl-xL (Chemnitz et al., 2004; Parry et al., 2005). The current study found disruption of PD-1 can increase the levels of Akt activation and anti-apoptotic protein Bcl-xL expression in GPC3-CAR T cells after combating the HCC cells, suggesting that the disruption of PD-1 can protect the GPC3-CAR T cell from exhaustion when combating the native PD-L1-expressing, GPC3-positive HCC. Among three analyzed tumor-related genes, *CTNNB1* and *MET* act as the oncogenes in HCC, and CCND1 is the hallmarker of cell cycle procession (Polakis, 2000; Zhang et al., 2002; Venepalli and Goff, 2013). Previous study found that HCC growth behavior was positively correlated with the expression levels of these tumor-related genes (Jiang et al., 2016). The present study found that the disruption of PD-1 can enhance the inhibition of the expression of tumor-related genes correlated with the HCC growth behavior caused by GPC3-CAR T cells, but the interaction mechanism between the PD-1 deficient GPC3-CAR T cells and HCC, and the influence of disruption of endogenous PD-1 on itself of GPC3-CAR T cells when combating tumor need the further in-depth studies by the combination of transcriptomics, proteomics, and bioinformatics, which will be beneficial for the design and development of the next-generation safe and more potent CAR T cells in HCC therapy.

## Conclusion

In summary, CRISPR-mediated disruption of endogenous PD-1 can enhance the CAR-dependent antitumor activity of the GPC3-specific second-generation CAR T cells employing CD28 as the co-stimulatory domain, and improve *in vivo* persistence and infiltration of CAR T cells, but not affect the CD4 and CD8 subsets, and activation status of CAR T cells. This study is beneficial for the development of next-generation CAR T cell with improved therapeutic efficacy in HCC by the precise genetic engineering.

## Author Contributions

ZL conceived the idea and revised the manuscript. XG designed subsequent experiments, performed most of the *in vitro* and *in vivo* work, and wrote the manuscript. HJ, BS, and MZ assisted with analysis of data and helped to perform the *in vitro* and *in vivo* work. HZ, ZS, GD, HL, XW, YW, and RS assisted with the *in vitro* work.

## Conflict of Interest Statement

Authors HZ, ZS, GD, and ZL were employed by company CARsgen Therapeutics, Shanghai, China. The remaining authors declare that the research was conducted in the absence of any commercial or financial relationships that could be construed as a potential conflict of interest. The reviewer HX and handling Editor declared their shared affiliation.

## Footnotes

1 http://crispr.mit.edu

## Abbreviations

CAR: chimeric antigen receptor
CRISPR/Cas: clustered regularly interspaced short palindromic repeat/CRISPR-associated protein
FBS: fetal bovine serum
GPC3: Glypican-3
GPC3-CAR: GPC3-specific 28ζ-CAR
gRNA: guide RNA
HCC: hepatocellular carcinoma
Indels: insertions or deletions
NSG: NOD-scid-IL-2Rγ-/-
PD-1: programmed death 1 receptor
PD-L: programmed death 1 ligand
UTD: untransduced T cells
