# Supplementary material for: Disruption of PD-1 Enhanced the Anti-tumor Activity of Chimeric Antigen Receptor T Cells Against Hepatocellular Carcinoma
Source: Front Pharmacol. 2018 Oct 1;9:1118. doi: 10.3389/fphar.2018.01118 (PMC6174208; doi:10.3389/fphar.2018.01118)
Supplement: Supplementary file 1 [file Data_Sheet_1.docx]

***Supplementary Material***

Disruption of PD-1 Enhanced the Anti-tumor Activity of Chimeric Antigen Receptor T cells against Hepatocellular Carcinoma

**Xingliang Guo****^1^, Hua Jiang^1^, Bizhi Shi****^1^, Min Zhou^1^, Honghong Zhang^2^, Zhimin Shi^2^, Guoxiu Du^2^, Hong Luo^1^, Xiuqi Wu^1^, Yi Wang^1^, Ruixin Sun^1^ and Zonghai Li^1,2*^**

^1^ State Key Laboratory of Oncogenes & Related Genes, Shanghai Cancer Institute, Renji Hospital, Shanghai Jiaotong University School of Medicine, Shanghai, China.

^2^ CARsgen Therapeutics, Shanghai, China.

***Correspondence:**

Prof. Li (Zonghai Li), PhD

No. 25/Ln 2200 Xie Tu Road, Shanghai, 200032, People’s Republic Of China.

Tel: 86-21-64436601;

Fax: 86-21-64432027;

Email: zonghaili@shsmu.edu.cn

**SUPPLEMENTARY FIGURE AND TABLES LEGENDS:**

**SUPPLEMENTARY FIGURE S1. PD-1 expression on the surface of wild-type GPC3-CAR T cells after expansion.** The PD-1 expression on the surface of wild-type GPC3-CAR T cells was measured by flow cytometry on day 9 post the activation of primary T cells with the anti-CD3/anti-CD28 beads.

**SUPPLEMENTARY FIGURE S2. Tumor weight of PLC/PRF/5 xenografts treated with the indicated T cells or PBS.** At the endpoint, the residual tumors of PLC/PRF/5 xenografts (n = 7) treated with PD-1-deficient GPC3-CAR T cells were significantly (***P* < 0.01) lighter than those treated with wild-type GPC3-CAR T cells. Data shown were mean ± SD from each treatment group. Bars, SD. ***P* < 0.01, and ****P* < 0.001 by one way ANOVA with Turkey post hoc test.

**SUPPLEMENTARY TABLE S1.** **Sequences of the templates for the *in vitro* transcription of gRNAs**

**SUPPLEMENTARY TABLE S2.** **Oligonucleotide primers used for the PCR amplification of off-target sites of CRISPR-mediated gene editing.**

**SUPPLEMENTARY TABLE S3.** **Oligonucleotide primers used for the sequencing of off-target sites of CRISPR-mediated gene editing.**

**SUPPLEMENTARY TABLE S4. Oligonucleotide primers used for the quantitative real-time PCR.**

**SUPPLEMENTARY TABLE S5. Analysis of off-target mutagenesis from the CRISPR-mediated gene editing.**


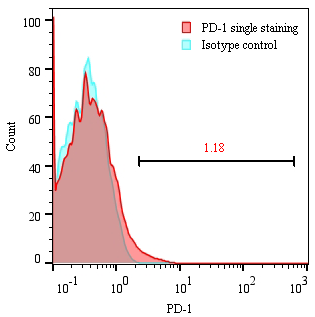


**SUPPLEMENTARY FIGURE S1. PD-1 expression on the surface of wild-type GPC3-CAR T cells after expansion.** The PD-1 expression on the surface of wild-type GPC3-CAR T cells was measured by flow cytometry on day 9 post the activation of primary T cells with the anti-CD3/anti-CD28 beads.

**
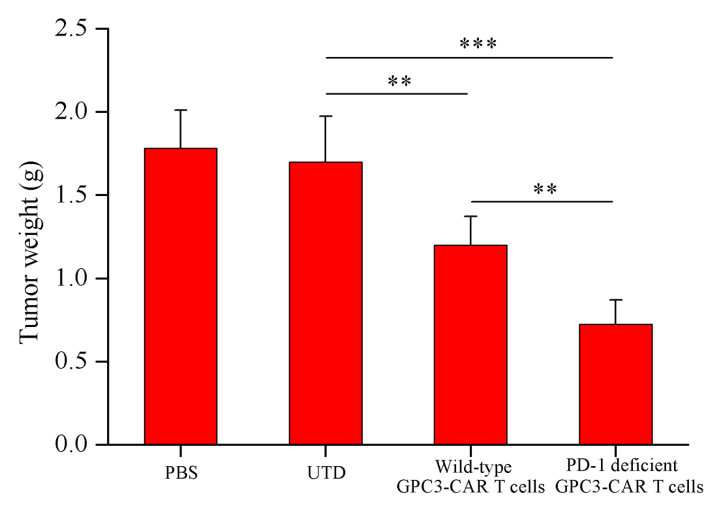
**

**SUPPLEMENTARY FIGURE S2. Tumor weight of PLC/PRF/5 xenografts treated with the indicated T cells or PBS.** At the endpoint, the residual tumors of PLC/PRF/5 xenografts (n = 7) treated with PD-1-deficient GPC3-CAR T cells were significantly (***P* < 0.01) lighter than those treated with wild-type GPC3-CAR T cells. Data shown were mean ± SD from each treatment group. Bars, SD. ***P* < 0.01, and ****P* < 0.001 by one way ANOVA with Turkey post hoc test.

**SUPPLEMENTARY TABLE S1.** **Sequences of the templates for the *in vitro* transcription of gRNAs**

| gRNA template | Sequence (5’ – 3’) |
| --- | --- |
| PD-1-gRNA-1-template | TAATACGACTCACTATAGGTCTGGGCGGTGCTACAACTGTTTTAGAGCTAGAAATAGCAAGTTAAAATAAGGCTAGTCCGTTATCAACTTGAAAAAGTGGCACCGAGTCGGTGCTTTTTTT |
| PD-1-gRNA-2-template | TAATACGACTCACTATAGGGCCAGGATGGTTCTTAGGTGTTTTAGAGCTAGAAATAGCAAGTTAAAATAAGGCTAGTCCGTTATCAACTTGAAAAAGTGGCACCGAGTCGGTGCTTTTTTT |

Red bases, T7 promoter; blue bases, target sequences; green bases, guide RNA scaffold.

**SUPPLEMENTARY TABLE S2.** **Oligonucleotide primers used for the PCR amplification of off-target sites of CRISPR-mediated gene editing.**

| Potential  off-target site | Primer name | Sequence (5’ – 3’) |
| --- | --- | --- |
| gRNA1-OT1 | gRNA1-OT1-FORWARD | GGGAGGGGAAGGGAGGAGGAGGGG |
|  | gRNA1-OT1-REVERSE | TGCCCAGCCTTGGACAGTTT |
| gRNA1-OT2 | gRNA1-OT2-FORWARD | ACTGCCATCAACCTTGCTTCC |
|  | gRNA1-OT2-REVERSE | CAGGTTGCCACTCCATTCAC |
| gRNA1-OT3 | gRNA1-OT3-FORWARD | CAGATGAGAGATCTGGGATGGAAA |
|  | gRNA1-OT3-REVERSE | ATGCCCACAGGTAGGTCAGG |
| gRNA1-OT4 | gRNA1-OT4-FORWARD | AACAAAAACTTAGATTTAAGA |
|  | gRNA1-OT4-REVERSE | CCACAAGTATTCCACCCAAC |
| gRNA1-OT5 | gRNA1-OT5-FORWARD | CACCCACCATTCCACTTTCT |
|  | gRNA1-OT5-REVERSE | CCTGCCATAGCCTCAGTTCC |
| gRNA2-OT1 | gRNA2-OT1-FORWARD | GGGAGGTGATGTCAAGATGTAGAGG |
|  | gRNA2-OT1-REVERSE | CAGCAAGCAGTGGGAGGGAC |
| gRNA2-OT2 | gRNA2-OT2-FORWARD | CTACCAGGAACCCAGGAAAGACC |
|  | gRNA2-OT2-REVERSE | CGTGGCGAGAACACTTTGGT |
| gRNA2-OT3 | gRNA2-OT3-FORWARD | TGGTGGCAAGCCCTGTCATA |
|  | gRNA2-OT3-REVERSE | TAAGACGTGCCTTTGCTTCC |
| gRNA2-OT4 | gRNA2-OT4-FORWARD | AAAAAGCTTTTTAAAAAATGAT |
|  | gRNA2-OT4-REVERSE | AAATCTGCTCCTCAAACTCA |
| gRNA2-OT5 | gRNA2-OT5-FORWARD | GGGACTACAGGTGCATGCCAC |
|  | gRNA2-OT5-REVERSE | GTAGGCTATTCACAAAGTTAGG |

OT, off-target site.

**SUPPLEMENTARY TABLE S3.** **Oligonucleotide primers used for the sequencing of off-target sites of CRISPR-mediated gene editing.**

| Potential  off-target site | Primer name | Sequence (5’ – 3’) |
| --- | --- | --- |
| gRNA1-OT1 | gRNA1-OT1-Seq-Forward | TGCTTCCATTTCCACCAGTTTCT |
| gRNA1-OT2 | gRNA1-OT2-Seq-Forward | GGCAGAGCCCTACTGGGAAAG |
| gRNA1-OT3 | gRNA1-OT3-Seq-Forward | TGAGCCATGTGGAACTGTAAGT |
| gRNA1-OT4 | gRNA1-OT4-Seq-Forward | GATCCATCCCAGCTTGACGT |
| gRNA1-OT5 | gRNA1-OT5-Seq-Forward | TCATTCGTCCCATAATTCTTTGAGC |
| gRNA2-OT1 | gRNA2-OT1-Seq-Forward | AACGGAACAGGTCTCCCTCACGC |
| gRNA2-OT2 | gRNA2-OT2-Seq-Forward | CAATAGTCCCTTGCCGATTCCA |
| gRNA2-OT3 | gRNA2-OT3-Seq-Forward | ACCCATCTAAGAATCAATGAAGCAC |
| gRNA2-OT4 | gRNA2-OT4-Seq-Forward | TAGGAATGTCTGAATTTGAAGGCACA |
| gRNA2-OT5 | gRNA2-OT5-Seq-Forward | CCTGAATGTATGTTTTGCTT |

OT, off-target site; Seq, sequencing.

**SUPPLEMENTARY TABLE S4. Oligonucleotide primers used for the quantitative real-time PCR**

| Gene | Primer name | Sequence (5’ – 3’) |
| --- | --- | --- |
| *Bcl-xL* | Bcl-xL-F | GGCTGGGATACTTTTGTGGA |
|  | Bcl-xL-R | GAAGAGTGAGCCCAGCAGAA |
| *CCND1* | CCND1-F | GGCGGATTGGAAATGAACTT |
|  | CCND1-R | TCCTCTCCAAAATGCCAGAG |
| *CTNNB1* | CTNNB1-F | ATTGTCCACGCTGGATTTTC |
|  | CTNNB1-R | AGGTCTGAGGAGCAGCTTCA |
| *MET* | MET-F | TGTTCGATATTCATCACGGC |
|  | MET-R | GCATTTTTACGGACCCAATC |
| *GAPDH* | GAPDH-F | ACCACAGTCCATGCCATCAC |
|  | GAPDH-R | TCCACCACCCTGTTGCTGTA |

*CCND1*, cyclin D1; *CTNNB1*, catenin beta 1; *MET*, MET proto-oncogene, receptor tyrosine kinase; *GAPDH*, glyceraldehyde 3-phosphate dehydrogenase.

**SUPPLEMENTARY TABLE S5. Analysis of off-target mutagenesis from the CRISPR-mediated gene editing.**

| Potential  off-target site | Locus in chromosome | Mismatch sequence (5’ – 3’) | Mutagenesis |
| --- | --- | --- | --- |
| gRNA1-OT1 | Chr6:-109541728 | CTGTGGGCAGTGCTACAACT | - |
| gRNA1-OT2 | Chr7:+140892224 | AACTGGGAGCTGCTACAACT | - |
| gRNA1-OT3 | Chr12:+106399029 | GTGTGGGTGCTGCTACAACA | - |
| gRNA1-OT4 | Chr8:-32001939 | TTCTGGCTAGTGCTACAACT | - |
| gRNA1-OT5 | Chr21:+33863413 | GCCTGGGAGATGGTACAACT | - |
| gRNA2-OT1 | Chr22:-30661978 | GGGCCGGATGGTTCTTAGGA | - |
| gRNA2-OT2 | Chr8:+133424641 | GTTCAGGATGGCTCTTAGGT | - |
| gRNA2-OT3 | Chr5:-88281429 | AGACTGGATAGTTCTTAGGT | - |
| gRNA2-OT4 | Chr7:+78358643 | GGTTAGACTGGTTCTTAGGT | - |
| gRNA2-OT5 | Chr6:+131596744 | AAGCAGGAGGGTTCTTAGGT | - |

Mutagenesis (Insertion and deletion) at the potential off-target sites were measured by TIDE analysis (n = 3). All TIDE analyses below the detection sensitivity of 1.5% were set to 0%. OT, off-target site; -, no mutation.
